# Correlation of KRAS G12C Mutation and High PD-L1 Expression with Clinical Outcome in NSCLC Patients Treated with Anti-PD1 Immunotherapy

**DOI:** 10.3390/jcm11061627

**Published:** 2022-03-15

**Authors:** Marco Cefalì, Samantha Epistolio, Giulia Ramelli, Dylan Mangan, Francesca Molinari, Vittoria Martin, Stefania Freguia, Luca Mazzucchelli, Patrizia Froesch, Milo Frattini, Luciano Wannesson

**Affiliations:** 1 Ente Ospedaliero Cantonale (EOC), Oncology Institute of Southern Switzerland, Via Ospedale 12, CH-6500 Bellinzona, Switzerland; d.man@manchester.ac.uk (D.M.); patrizia.froesch@eoc.ch (P.F.); luciano.wannesson@eoc.ch (L.W.); 2 Ente Ospedaliero Cantonale (EOC), Institute of Pathology, Via in Selva 24, CH-6601 Locarno, Switzerland; samantha.epistolio@eoc.ch (S.E.); giulia.ramelli@eoc.ch (G.R.); francesca.molinari@eoc.ch (F.M.); vittoria.martin@eoc.ch (V.M.); stefania.freguia@eoc.ch (S.F.); luca.mazzucchelli@eoc.ch (L.M.); milo.frattini@eoc.ch (M.F.)

**Keywords:** KRAS mutation, immune checkpoint inhibitor (ICI), progression-free survival (PFS), overall survival (OS), non-small cell lung cancer (NSCLC), PD-L1

## Abstract

Background: Immune checkpoint inhibitors (ICIs) targeting PD-1 or PD-L1 improved the survival of non-small cell lung cancer (NSCLC) patients with PD-L1 expression ≥50% and without alterations in EGFR, ALK, ROS1, RET. However, markers able to predict the efficacy of ICIs, in combination with PD-L1 expression are still lacking. Our aim in this hypothesis-generating pilot study was to evaluate whether the KRAS G12C variant may predict the efficacy of ICIs in advanced NSCLC patients with PD-L1 ≥ 50%. Methods: Genomic DNA or tissue sections of 44 advanced ICI-treated NSCLC cases with PD-L1 ≥ 50% without EGFR, ALK, ROS1, RET alterations were tested using Next Generation Sequencing, Fluorescence in Situ Hybridization and immunohistochemistry. Statistical analyses were carried out fitting univariate and multivariate time to event models. Results: KRAS G12C mutant patients (N = 11/44) showed a significantly longer progression-free survival (PFS) at univariate and multivariate analyses (*p* = 0.03). The Kaplan–Meier plot of the PFS time-to-event supports that G12C positive patients have a longer time to progress. PFS improvement was not observed when any KRAS mutations were compared to wild-type cases. Conclusions: Given the limitations due to the small sample size and exploratory nature of this study, we tentatively conclude the KRAS G12C mutation should be considered in future trials as a predictive marker of prolonged response to first-line ICIs in NSCLC patients overexpressing PD-L1. This finding could be relevant as anti-KRAS G12C therapies enter the therapeutic landscape of NSCLC.

## 1. Introduction

The treatment landscape of advanced non-small cell lung cancer (NSCLC) has significantly evolved in recent decades, with the advent of molecularly targeted agents and immunotherapy [1]. The choice of first-line treatment is primarily based on the identification of actionable oncogenic drivers, (such as EGFR mutations, and ALK, ROS and RET translocations) and, in their absence, on the presence of biomarkers predictive of immunotherapeutic response, such as programmed death ligand 1 (PD-L1), together with other clinical and pathological characteristics [2,3].

Before the age of immunotherapy, patients whose disease could not benefit from targeted treatment with tyrosine kinase inhibitors were assigned by default to standard platinum-based chemotherapy, in the absence of contraindications [4]. With the introduction of immune checkpoint inhibitors (ICIs), particularly following data from the phase III KEYNOTE-024 trial, new treatment options have opened up for the subgroup of patients without actionable mutations and with PD-L1 expression greater than or equal to 50% [5]. Indeed, the patients with these characteristics are eligible for first-line treatment with the ICI pembrolizumab.

Despite the absence of a direct comparison of immunotherapy alone to immunotherapy combined with a platinum-based doublet, cross-trial comparisons from the KEYNOTE studies 024,189,407 and IMpower 110 and 132, seem to establish that survival outcomes are similar regardless of the addition of chemotherapy to the ICIs in the PD-L1 ≥ 50% subgroup. However, ample differences in toxicity, in favor of single-agent immunotherapy, have been reported and as a consequence, pembrolizumab monotherapy has been proposed as a standard first-line option in ESMO guidelines [5,6,7,8,9,10]. A subset of patients without actionable mutations, with PD-L1 expression of at least 50%, treated according to this rationale, achieves exceptionally sustained responses. Attempts have been made to identify predictive biomarkers for this favorable outcome, however, despite emerging data concerning tumor mutational burden and somatic copy number alterations, only PD-L1 expression is routinely employed in clinical practice [11,12], although with limited possibility to clearly predict the response to ICIs.

It has long been known, however, that the presence of specific driver mutations can influence tumor sensitivity to immunotherapy; the most evident example lies in the fact that the activity of ICIs is significantly reduced in disease harboring EGFR, ALK, or ROS1 alterations, which justifies tyrosine kinase inhibitor treatment being prescribed in the first line instead of immunotherapy [12,13]. It is intuitive that first-line therapy can be guaranteed for a larger number of lung cancer patients compared to second-line treatment. This phenomenon is due to clinical deterioration or death, causing a drop-out (“attrition”) of more than 50% between treatment lines. For this reason, it is essential to administer the most effective treatment first [14,15,16]. While new agents directed against emerging targets such as BRAF V600, KRAS G12C, MET, RET, NTRK and HER2 keep being developed, the prognostic and predictive role of these new alterations has not entirely been clarified. According to recent data, some NSCLC patients with alterations in BRAF, MET, RET or HER2 may benefit from ICIs in a similar way as unselected NSCLC patients, but larger prospective studies are needed [17].

Recent literature has suggested an association between the presence of mutations in the KRAS gene and improved benefit from first-line immunotherapy; for instance, Sun et al. found that overall survival (OS) appeared to differ, in favor of patients treated with first-line immunotherapy combined with chemotherapy, rather than immunotherapy alone, in KRAS wild type (wt) tumors, but not in KRAS mutant tumors [18]. A meta-analysis by Lee et al. reported that ICIs appeared to improve survival compared to docetaxel, in the subgroup of KRAS mutant patients but not in KRAS wt ones [19]. Another study by Dong et al., also suggested an increased clinical benefit from immunotherapy in patients whose disease carried mutations in KRAS or TP53, although these results were not always confirmed in other studies [20,21,22,23].

The recently developed KRAS G12C inhibitor sotorasib (AMG510), which achieves response rates of up to 40% in pretreated patients, is posited to become an extremely important therapy for the treatment of this subpopulation of tumors; consequently, it would be important for the choice of first-line strategy to determine what the response rate and survival perspective with first-line immunotherapy is for the subgroup of patients with high PD-L1 expression and a concomitant KRAS G12C mutation [24].

As discussed above, prior observations trying to correlate immunotherapy in general (with or without chemotherapy, at any level of PD-L1 expression) with KRAS mutations failed to find robust interactions. For this reason, we focused our attention on the PD-L1 ≥50% population treated in the first line with ICI alone, in order to narrow biological variability and to obtain a more homogeneous population. With similar intent, we also differentiated between KRAS G12C vs non-G12C. The availability of a G12C inhibitor makes such an analysis potentially helpful to guide further research on the best sequencing strategy.

## 2. Materials and Methods

### 2.1. Patients

We identified 44 patients with advanced or metastatic NSCLC with high PD-L1 expression (≥50%), treated at our institution with first-line immunotherapy. Following evaluation and approval from the local Ethics Committee, we searched our records for clinical data concerning age, gender, date of diagnosis of advanced disease and stage, ECOG performance status, smoking status, presence or absence of central nervous system metastases, presence or absence of grade 3 or higher immune-related toxicities, best response to immunotherapy evaluated by RECIST, date of disease progression, date of last follow-up or death.

We also gathered pathology data concerning histology, PD-L1 expression, and molecular biology analyses which are routinely performed in patients with a diagnosis of advanced NSCLC; specifically, the identification of mutations or gene fusions in EGFR, ALK, ROS, KRAS, NRAS, BRAF, STK11, TP53, PIK3CA, HER2, MET, RET.

Patients whose disease carried alterations that made them candidates for targeted therapy in the first line, thus specifically in EGFR, ALK, ROS1 or RET, were not included in the search because, according to usual clinical practice, they did not receive first-line immunotherapy.

### 2.2. Mutational Analyses in Tissue Samples

The molecular characterization of all the samples was performed by applying three methodologies: next-generation sequencing for the identification of point mutations and small insertions or deletions, Fluorescence in Situ Hybridization (FISH) for gene fusions, and Immunohistochemistry for protein expression. For these analyses, different slide thicknesses of the same Formalin-Fixed Paraffin Embedded (FFPE) tissue block were cut. The tumor specimen and area were selected by a pathologist of the Institute of Pathology in Locarno (Switzerland), who specialized in lung cancer.

#### 2.2.1. Next-Generation Sequencing Analyses

Genomic DNA extraction was performed following the QIAamp DNA FFPE Tissue kit (Qiagen, Chatsworth, CA, USA) manufacturer‘s instructions starting from three 8 µm-thick serial sections of the selected FFPE tissue block. The extracted genomic DNA was characterized through a next-generation sequencing approach on the S5XL Ion Torrent platform, using the Ion Ampliseq Colon and Lung Cancer Panel v2 (CLv2) panel (ThermoFisher Scientific, Waltham, MA, USA).

The CLv2 panel provides data about the mutational status of 22 genes (92 hot spot regions), including the most relevant and frequently mutated genes in lung adenocarcinoma (i.e.: AKT1, ALK, BRAF, CTNNB1, DDR2, EGFR, ERBB2, ERBB4, FBXW7, FGFR1, FGFR2, FGFR3, KRAS, MAP2K1, MET, NOTCH1, NRAS, PIK3CA, PTEN, SMAD4, STK11, TP53).

Ion Torrent results were considered evaluable only when the target regions were covered at least by 500 reads, the mean depth values were greater than 1100X and the coverage uniformity was greater than 90%. The limit of detection for single nucleotide variant identification was set at 2–5% but it could be influenced by different factors such as tumor cellularity, acid nucleic degradation or some interferences at the DNA amplification level. All the mutations known in the literature for being benign variants/polymorphisms and all the variants included in intronic regions (with the exception of untranslated regions or splice site regions), were excluded.

#### 2.2.2. Fluorescence In Situ Hybridization

FISH was performed on 4-μm FFPE tissue sections treated with the Paraffin Pretreatment kit II (Pretreatment Reagent VP 2000, Abbott Molecular AG; Baar, Switzerland) and processed with the VP2000 automatic processor (Abbott Molecular AG) according to the manufacturer’s instructions.

The presence of chromosomal alterations (gene fusions) in ALK, ROS1 and RET were evaluated using different probes: the ALK Break Apart FISH Probe Kit (Abbott Molecular Vysis; North Chicago, IL, USA), the SPEC ROS1 Dual Color Break Apart Probe (Zytovision; Bremerhaven, Germany) and the Zytolight SPEC RET Dual Color Break Apart Probe (Zytovision), respectively.

The evaluation of FISH results was performed following the criteria previously published [25,26,27]. As for interpretation criteria, a minimum of 100 morphologically clear, non-overlapping nuclei from at least 8–10 areas were scored for each patient. Only experiments with at least 90% hybridization efficiency were considered. A cut-off of 15% was applied to consider a tissue positive for ALK, ROS1 and RET rearrangement.

#### 2.2.3. Immunohistochemistry

The immunohistochemical evaluation of PD-L1, aimed to evaluate its protein expression, was performed using the SP263 monoclonal rabbit anti-human antibody (Ventana/Roche, Ventana Medical Systems) on an automated instrument (Benchmark GX, Ventana/Roche), according to the standard protocol. The tumor proportion score was applied [28].

### 2.3. Statistical Analysis

The endpoints of progression-free survival (PFS) and OS were the measures in consideration. The study population was divided into one of the following two groups: patients whose tumors harbored a KRAS G12C mutation (G12C+) and patients without KRAS G12C mutated disease (G12C−). Additional comparisons were made between patients with any KRAS mutation (KRAS+) and those with no KRAS mutation (KRAS−), in order to better highlight the specific relevance of the G12C mutation.

The balance between the G12C+/− groups was assessed among relevant factors available for the study. A univariate Kaplan–Meier analysis with the log-rank test for significance was then undertaken, followed by a multivariate Cox Proportional Hazard model including: a patient’s G12C+/− or KRAS+/− status, ECOG performance status at initiation of immunotherapy, status as a current or former smoker, and age at diagnosis of advanced disease. This was a retrospective study, and no sample size calculation was used, in part due to the lack of prior analyses indicating an expected effect size. Instead, all available data from our institution was used, and a number of covariates for our multivariate model were selected according to the rule of thumb of 10 observations per covariate. For Cox Proportional Hazard models, possible highly influential points were checked using deviance residuals and the proportional hazard assumption using Schoenfeld residuals [29]. The Akaike information criterion [30] was used to test for overfitting in models, especially important due to the small sample size. All analysis was conducted in R version 4.0.3 [31], in RStudio build 351 [32], with additional packages: readxl [33] survival [34], dplyr [35], ggplot2 [36], ggfortify [37] and survminer [38]. Fisher exact tests [39] were used to give an idea of whether differences in the frequencies of events between those with a KRAS G12C mutation and those with any other KRAS mutation, such as the small sample size, meant this was the only direct comparison test possible.

## 3. Results

In our cohort, characterized by the absence of EGFR, ALK, ROS1 and RET alterations (as per exclusion criteria related to upfront treatment with ICI), a KRAS mutation was identified in 25 patients, corresponding to 57% of the population. As expected, the G12C variant was the most frequent mutation, accounting for 25% of the whole cohort and 44% of the KRAS mutant patients (Supplementary Table S1).

Patients were divided into the following two groups: a cohort whose tumors harbored a KRAS G12C mutation and a cohort without KRAS G12C mutated disease. Characteristics of these two groups are displayed in Table 1.

**Figure 1:**
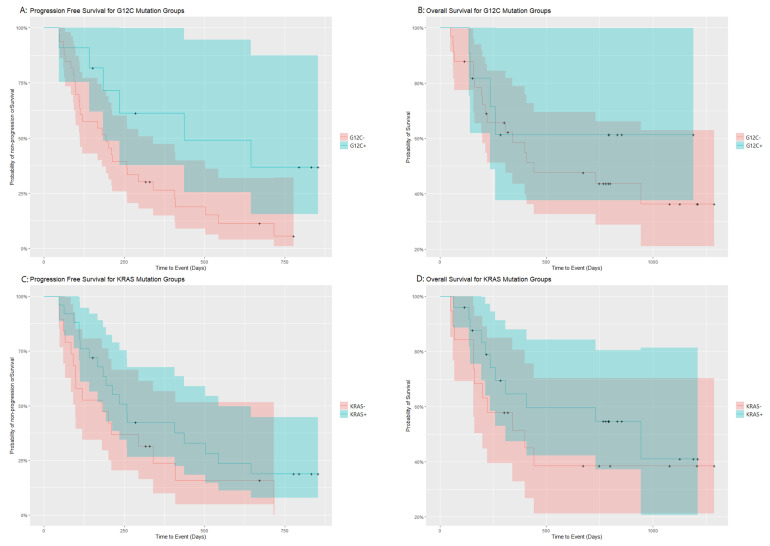
Kaplan–Maier curves of overall survival and progression-free survival split by G12C status and KRAS mutation status.

The PFS and OS of these two groups were analyzed using the log rank test. Table 2 provides a summary of PFS and OS events in the study population, for both the KRAS G12C+/− and the KRAS+/− analysis. With a median follow–up of 11 months, the G12C mutant group showed a statistically significantly better median PFS (14.6 months, 95% CI 6.1-incalculable; compared to 6.5 months 95% CI 3.7–11.3; χ2(1) = 4.5 *p* = 0.03); the relative Kaplan–Meier plot is reported in Figure 1A. Overlapping confidence intervals are not necessarily an indication that the significant effect should be disregarded, only an indication of where we should expect most studies would find the median survival of the groups [40]. Furthermore, in our case, confidence intervals are not a reliable metric due to the small sample size and outliers, which are discussed later in the article. No trend toward better OS was observed, median OS incalculable (95% CI 7.9-incalculable) in the G12C+ group, compared to 14.7 months (95% CI 10.2-incalculable), without statistical significance χ2(1) = 0.5 *p* = 0.47, made especially difficult by the low number of events for OS in the G12C mutant group (Figure 1B).

To better evaluate the role of the G12C variant as a prognostic factor for PFS, a Cox proportional hazard model was fitted, including a patient’s G12C mutation status (positive or negative), ECOG performance status at initiation of immunotherapy, status as a current or former smoker, and age at diagnosis of advanced disease. The balance of these secondary covariates was checked with regard to the primary variable of interest, G12C status, as per Table 1; no variables required the model to be weighted.

The model was not significant overall (likelihood ratio χ2(4) = 5.49, *p* = 0.2, log rank χ2(4) = 5.07 *p* = 0.2), nor with respect to any of the individual variables, specifically G12C+ giving a hazard ratio of 0.42 (95% CI 0.16–1.08, *p* = 0.07). The other covariates were: ECOG HR 1.01, (95% CI 0.68–1.50, *p* = 0.95), Past/Current smoker HR 0.63, (95% CI 0.14–2.85, *p* = 0.55), Presence of brain metastases HR 1.12, (95% CI 0.63–1.97, *p* = 0.70). Inspection of Schoenfeld residuals did not indicate a violation of the proportional hazards assumption. Inspection of deviance residuals, however, identified four patients where |e| > 2, suggesting a potentially exaggerated effect on the model, which is of special relevance due to the small sample size. Pointwise deletion led to significant changes to the model, therefore all these patients were removed from the analysis, according to this rule of thumb, producing a second model, which was significant overall (likelihood ratio χ2(4) =1 0.73 *p*= 0.03, log rank χ2(4) = 9.58 *p* = 0.05), indicating a significant predictive association of G12C+ status to PFS with a hazard ratio of 0.27 (95% CI 0.1–0.76, *p* = 0.01). The other covariates were: ECOG HR 1.14, (95% CI 0.68–1.92, *p* = 0.61), Past/Current smoker HR 0.62, (95% CI 0.14–2.85, *p* = 0.54), Presence of brain metastases HR 1.08, (95% CI 0.63–1.86, *p* = 0.78). This second model also did not violate the proportional hazards assumption. Both these sets of results along with model level measures and hazard ratios, confidence intervals and *p* values of individual covariates are summarized in Table 3. Akaike information criterion estimation was also used to assess both these models, which are likely over-fit as in both cases the conclusion was to remove all factors except G12C mutant status from the model resulting in both a significant model and highlighting G12C mutation as being a significant predictor of improved PFS; model 1 having likelihood ratio test χ2(1) = 5.07 *p* = 0.02 and log rank χ2(1) = 4.53 *p* = 0.03; G12C+ HR 0.39, (95% CI 0.16–0.96, *p* = 0.04). Model 2 having likelihood ratio test χ2(1) = 10.18 *p* < 0.01 and log rank χ2(1) = 8.88 *p* < 0.01, G12C+ HR 0.24 (95% CI 0.09–0.66, *p* = 0.01). Full details are reported in Supplementary Table S2 for completeness.

Whilst it would be improper to conclude that a G12C mutation confers a survival advantage in this group of patients, the significant effect indicated in model 2, alongside the tentative result of the univariate analysis of G12C mutated patients, are indicators that this may be the case.

A further exploratory analysis was carried out, comparing PFS in patients with any KRAS mutation versus patients with wt KRAS gene status. The trend towards better PFS in the KRAS mutated subgroup was not statistically significant (log rank χ2(1) = 1.8 *p* = 0.18), suggesting that the improvement in outcomes is not tied to the presence of any KRAS mutation, but specifically to G12C, although data from a larger dataset are required to confirm this hypothesis. These groups were also not significant when considering OS, χ2(1) = 0.9 *p* = 0.35. The Kaplan-Meier plots relative to these analyses are reported in Figure 1C,D, the almost completely overlapping confidence interval bands plotted, further supporting the argument for no significant difference between the groups.

A Cox proportional hazard model was also fitted for this comparison, with KRAS mutational status as the primary variable of interest and ECOG performance status, status as a current or former smoker, presence of brain metastases, and age at diagnosis as secondary covariates. In analogy to the analysis carried out with G12C status as primary variable, the original model was not significant, with likelihood ratio χ2(4) = 3.98 *p* = 0.4 and log rank χ2(4) = 4.17 *p* = 0.4, KRAS+ HR 0.6 (95%CI 0.30–1.18, *p* = 0.14). The other covariates were: ECOG HR 1.16, (95% CI 0.78–1.71, *p* = 0.46), past/current smoker HR 0.43, (95% CI 0.10–1.88, *p* = 0.26), presence of brain metastases HR 1.37, (95% CI 0.79–2.36, *p* = 0.26). Subsequent iterations were carried out first through outlier removal leading to model 2 with likelihood ratio χ2(4) = 6.64 *p* = 0.2 and log rank χ2(4) = 7.1 *p* = 0.1; KRAS+ HR 0.46, 95% CI 0.22–0.95, *p* = 0.04. With the additional covariates: ECOG HR 1.04, (95% CI 0.68–1.58, *p* = 0.85), past/current smoker HR 0.39, (95% CI 0.09–1.72, *p* = 0.21), presence of brain metastases HR 1.61, (95% CI 0.93–2.80, *p* = 0.09). Then, Akaike information criterion estimation was used to select models with more optimized sets of covariates; model 1 gave likelihood ratio test χ2(1) = 1.75 *p* = 0.2 and log rank test χ2(1) = 1.82 *p* = 0.2, KRAS+, the only covariate indicated to remain in the model, had HR 0.63 95%CI 0.32–1.24, *p* = 0.18. The second optimized model had likelihood ratio χ2(2) = 5.4 *p* = 0.07 and log rank χ2(2) = 5.72 *p* = 0.06, KRAS+ HR 0.47 (95% CI 0.23–0.97, *p* = 0.04) and presence of brain metastases HR 1.55 (95% CI 0.90–2.69 *p* = 0.11). These statistics indicate none of these models as significant, as reported in Table 4 and Supplementary Table S3. Schoenfeld residuals were calculated and were non-significant for all these models, meaning the proportional hazards assumption was not being violated by any of these models.

Even though from a biological perspective, activating mutations would be expected to have similar effects irrespective of the specific mutation, the KRAS models at most only showed a trend for the totality of KRAS mutations in predicting a reduced risk of a PFS event, remaining non-significant overall. This could either be a consequence of a small sample size or may imply that the beneficial survival effect is driven only by the G12C mutated subgroup of KRAS mutations.

The sample size was not sufficient to compare G12C mutated and KRAS mutated cases excluding G12C patients using advanced statistical methods, however, Fisher’s exact *p* values were calculated comparing the number of events for PFS, indicating a significant difference (*p* = 0.03) with a lower proportion of events in the G12C mutated group than in other KRAS mutations (Table 5).

## 4. Discussion

The evaluation of PD-L1 is used to identify NSCLC patients who can be treated with single-agent immunotherapy initially [5,6,7,8,9,10]. However, the benefit derived from this treatment is highly variable, leading to a need for the identification of new predictive biomarkers. We focused our attention on the molecular alteration that is prevalent in the development of NSCLC, i.e., KRAS gene mutations. With the limitations of a small sample size and retrospective design, our observations point towards a more significant benefit from immunotherapy in patients whose tumors specifically harbor the KRAS G12C mutation. A statistically significant association in the same direction was not observed when taking into account any KRAS mutation, possibly due to the small sample size of our cohort. However, this finding suggests that the role played by KRAS mutations in the context of prolonged survival in NSCLC patients with PD-L1 overexpression should potentially be attributed to the G12C variant.

The finding that specific mutated alleles of a single gene may impact immunotherapy efficacy in different manners is not unexpected; for instance, in a 2019 retrospective study, it was observed that outcomes of PD-L1 blockade were worse in NSCLC patients harboring mutations in exon 19 of EGFR, but not with the L858R variant in exon 21, compared with EGFR wt patients. In addition, although a biological rationale for this has not yet been established, the same study found that exon 19 mutations appeared to correlate with a lower tumor mutational burden than L858R mutations [41].

KRAS mutations may well behave in a similar fashion, although our study was not powered to evaluate the differential impact of KRAS mutation variants, nor their interactions with other mutations such as STK11 or TP53.

Again, we stress that further prospective analysis is required to validate any conclusions drawn from our data, which should be seen as hypothesis-generating. It is also important to highlight the limited applicability of these findings to only the patient group characterized by PD-L1 overexpression, along with the other criteria used to hone in on this specific group of interest. The small number of events in some groups of interest further compounded the difficulties of the small sample size and the potential sensitivity of the model has been highlighted by the changes given through pointwise deletion of influential points. An alternative way of addressing these was not possible, however, due to the small sample sizes, so instead we have clearly given models both pre- and post-deletion; in a larger cohort, more rigorous methods for handling outliers would lead to a more stable conclusion. A larger sample would also facilitate the direct comparison of G12C+ patients to those with other types of KRAS mutation. All our findings need to be validated in larger sample studies, ideally controlling for additional covariates; this study however provides preliminary results and thereby a basis for the planning of these studies.

The KRAS G12C mutation is of particular interest due to the fact that a specific inhibitor with proven clinical activity exists (sotorasib), but its role in the therapeutic landscape is yet to be defined [24]. It also remains to be determined whether the standard first-line treatment of NSCLC harboring patients with a KRAS G12C mutation will continue to be immunotherapy (in the group showing PD-L1 expression ≥50%), or if it would make sense to explore a sotorasib-first strategy in future clinical trials.

As discussed, patient attrition between treatment lines makes the issue of therapeutic sequencing a relevant one in terms of survival outcomes. If larger studies confirm this suggested trend towards better outcomes with immunotherapy in KRAS G12C, PD-L1 overexpressed (≥50%) NSCLC, it would be logical to explore the upfront use of sotorasib in PD-L1 low/negative, KRAS G12C NSCLC compared to the actual standard of chemo-immunotherapy [6,24]; while the PD-L1 high subgroup would continue to be treated with ICIs in the first-line, given the relatively high likelihood of a durable response.

## 5. Conclusions

The introduction of an increasingly broad spectrum of therapies creates new opportunities, but also challenges because the ideal sequence of new treatment administration must be determined. Identifying subgroups that are more or less likely to benefit from a certain sequencing strategy can assist in orienting the design of future trials and thus begin mapping the ever-expanding landscape of NSCLC treatment, with the objective of maximizing the patient benefit from pharmacological advancement.

In conclusion, our data potentially indicates an expanded clinical role of the KRAS G12C variant, not only as a target for a new tailored treatment, but also in the identification of patients who may benefit most from ICI first-line treatment.

## Figures and Tables

**Table 1:** Summary of Patient’s Characteristics. Abbreviations: G12C+, cases with a G12C mutation in the KRAS gene; G12C−, cases without a G12C mutation in the KRAS gene; mut, mutation; *N*, number; NA, not available, *p*, *p* value.

Factor (%)	Entire Sample	G12C+	G12C−	Statistical Test between Groups to Assess Balance
*N*	44	11 (25%)	33 (75%)	NA
Number of Females	19 (43%)	4 (21%)	15 (79%)	Fisher’s Exact *p* = 0.73
Presence of Brain Metastases	11 (25%)	2 (18%)	9 (27%)	Fisher’s Exact *p* = 0.70
Smokers, actual or former	42 (95%)	11 (100%)	31 (94%)	Fisher’s Exact *p* = 1
Median Age	69	71	68	H(1) = 1.43, *p* = 0.23
Median ECOG	1	1	1	H(1) = 0.68, *p* = 0.68

**Table 2:** Summary of PFS and OS Events in the Study Population. Abbreviations: G12C+, cases with G12C mutations in KRAS gene; G12C−, cases without a G12C mutation in the KRAS gene; KRAS+, patients with KRAS mutations; KRAS−, patients without KRAS mutation; mut, mutations; OS, overall survival; *p*, *p* value; PFS, progression-free survival.

Group	Progression Free Survival			Overall Survival		
	Events	Median Time to Event (Days)	95% CI (Days)	Events	Median Time to Event (Days)	95% CI (Days)
Entire Population	35	211	168–407	22	731	307-NA
KRAS+	19	258	98-NA	11	944	307-NA
KRAS−	16	181	184–544	11	398	200-NA
Log rank test	χ2(1) = 1.8 *p* = 0.18			χ2(1) = 0.9 *p* = 0.35		
G12C+	6	437	184-NA	4	NA	236-NA
G12C−	29	194	112–340	18	442	307-NA
Log rank test	χ2(1) = 4.5 *p* = 0.03			χ2(1) = 0.5 *p* = 0.47		

**Table 3:** Description of Cox Proportional Hazard Models (PFS) for G12C+/−. Abbreviations: CI, confidence interval; G12C+, patients with G12C mutation in *KRAS* gene; *p*, *p* value. Statistically significant *p* values are shown in bold.

Risk Factor	Model 1			Model 2		
	Hazard Ratio	95% CI	*p*	Hazard Ratio	95% CI	*p*
G12C+	0.42	0.16–1.08	0.07	0.27	0.10–0.76	**0.01**
ECOG	1.01	0.68–1.50	0.95	1.14	0.68–1.92	0.61
Past/current Smoker	0.63	0.14–2.85	0.55	0.62	0.14–2.85	0.54
Presence of brain metastases	1.12	0.63–1.97	0.70	1.08	0.63–1.86	0.78
Likelihood ratio test	χ2(4) = 5.49		0.2	χ2(4) = 10.73		**0.03**
Wald Test	χ2(4) = 4.74		0.3	χ2(4) = 8.44		0.08
Log Rank Test	χ2(4) = 5.07		0.2	χ2(4) = 9.58		0.05
Concordance	0.59			0.65		

**Table 4:** Description of Cox Proportional Hazard Models (PFS) for KRAS+/−. Abbreviations: CI, confidence interval; KRAS+, patients with any mutation in KRAS gene; *p*, *p* value.

Risk Factor	Model 1			Model 2		
	Hazard Ratio	95% CI	*p*	Hazard Ratio	95% CI	*p*
KRAS+	0.60	0.30–1.18	0.14	0.46	0.22–0.95	0.04
ECOG	1.16	0.78–1.71	0.46	1.04	0.68–1.58	0.85
Past/current Smoker	0.43	0.10–1.88	0.26	0.39	0.09–1.72	0.21
Presence of brain metastases	1.37	0.79–2.36	0.26	1.61	0.93–2.80	0.09
Likelihood ratio test	χ2(4) = 3.98		0.4	χ2(4) = 6.64		0.2
Wald Test	χ2(4) = 4.1		0.4	χ2(4) = 6.8		0.1
Log Rank Test	χ2(4) = 4.17		0.4	χ2(4) = 7.1		0.1
Concordance	0.59			0.62		

**Table 5:** Summary of PFS events in the KRAS mutation group divided by G12C mutation and other KRAS mutations. Abbreviations: KRAS G12C+, cases with G12C mutations in KRAS gene; mut, mutations; *p*, *p* value; PFS, progression-free survival. Statistically significant *p* values are shown in bold.

Group	PFS Events	
	No Event	Event
*KRAS* G12C+	5	6
Other *KRAS* mut	1	13
Fisher’s Exact *p*	**0.03**	

## Data Availability

The datasets used and analyzed during the current study are available from the corresponding author on reasonable request. The data are not publicly available due to institutional policy.

## Supplementary Materials

The following supporting information can be downloaded at: https://www.mdpi.com/article/10.3390/jcm11061627/s1, Table S1: Description of KRAS mutational status. Abbreviations: *N*, number of cases; wt, wild type, Table S2: Description of AIC Optimised Cox Proportional Hazard Models for G12C+/−. Abbreviations: CI, confidence interval; G12C+, patients with G12C mutation in KRAS gene; *p*, *p* value, Table S3: Description of AIC Optimised Cox Proportional Hazard Models for KRAS+/−. Abbreviations: CI, confidence interval; KRAS+, patients with any mutation in KRAS gene; *p*, *p* value.
