# Supplementary material for: Correlation of KRAS G12C Mutation and High PD-L1 Expression with Clinical Outcome in NSCLC Patients Treated with Anti-PD1 Immunotherapy
Source: J Clin Med. 2022 Mar 15;11(6):1627. doi: 10.3390/jcm11061627 (PMC8954500; doi:10.3390/jcm11061627)
Supplement: Supplementary file 1 [file jcm-11-01627-s001.zip › jcm-1589291-supplementary.pdf]

Table S1. Description of KRAS mutational status. Abbreviations: *N*, number of cases; wt, wild type.

	Entire sample	G12C	G12A	G12D	G12R	G12S	G12V	G13D	wt
N (%)	44 (100%)	11 (25%)	1 (2%)	5 (11%)	1 (2%)	1 (2%)	5 (11%)	1 (2%)	19

Table S2. Description of AIC Optimised Cox Proportional Hazard Models for G12C+/-. Abbreviations: CI, confidence interval; G12C+, patients with G12C mutation in *KRAS* gene; *p*, *p* value.

Risk Factor	Model 1			Model 2		
	Hazard Ratio	95% CI	*p*	Hazard Ratio	95% CI	*p*
G12C+	0.39	0.16-0.96	0.04	0.24	0.09-0.66	0.01
Likelihood ratio test	$\chi2(1)=5.07$		0.02	$\chi2(1)=10.18$		<0.01
Wald Test	$\chi2(1)=4.24$		0.04	$\chi2(1)=7.82$		0.01
Log Rank Test	$\chi2(1)=4.53$		0.03	$\chi2(1)=8.88$		<0.01
Concordance	0.58			0.62		

Table S3. Description of AIC Optimised Cox Proportional Hazard Models for *KRAS*+/-. Abbreviations: CI, confidence interval; *KRAS*+, patients with any mutation in *KRAS* gene; *p*, *p* value.

Risk Factor	Model 1			Model 2		
	Hazard Ratio	95% CI	*p*	Hazard Ratio	95% CI	*p*
KRAS+	0.63	0.32-1.24	0.18	0.47	0.23-0.97	0.04
Presence of brain metastases				1.55	0.90-2.69	0.11
Likelihood ratio test	$\chi2(1)=1.75$		0.2	$\chi2(2)=5.4$		0.07
Wald Test	$\chi2(1)=1.79$		0.2	$\chi2(2)=5.2$		0.06
Log Rank Test	$\chi2(1)=1.82$		0.2	$\chi2(2)=5.72$		0.06
Concordance	0.57			0.62		
